# The impact of a multidisciplinary self-care management program on quality of life, self-care, adherence to anti-hypertensive therapy, glycemic control, and renal function in diabetic kidney disease: A Cross-over Study Protocol

**DOI:** 10.1186/s12882-016-0279-6

**Published:** 2016-07-19

**Authors:** Nancy Helou, Dominique Talhouedec, Maya Shaha, Anne Zanchi

**Affiliations:** HESAV, The University of Health Sciences (HES-SO), Av. de Beaumont 21, 1011 Lausanne, Switzerland; Faculty of Biology and Medicine, University Institute of Higher Education and Research in Healthcare (IUFRS), University of Lausanne, Biopôle 2, Route de la Corniche 10, 1010 Lausanne, Switzerland; Services of Nephrology, Diabetes and Endocrinology, Department of Internal Medicine, Centre Hospitalier Universitaire Vaudois (CHUV), Rue du Bugnon 17, 1011 Lausanne, Switzerland; Clinique de La Source, Avenue Vinet 30, 1004 Lausanne, Switzerland

**Keywords:** Diabetic nephropathies, Diabetic nephropathy, Interdisciplinary studies, Multidisciplinary care, Self-care

## Abstract

**Background:**

Diabetic kidney disease, a global health issue, remains associated with high morbidity and mortality. Previous research has shown that multidisciplinary management of chronic disease can improve patient outcomes. The effect of multidisciplinary self-care management on quality of life and renal function of patients with diabetic kidney disease has not yet been well established.

**Method/Design:**

The aim of this study is to evaluate the impact of a multidisciplinary self-care management program on quality of life, self-care behavior, adherence to anti-hypertensive treatment, glycemic control, and renal function of adults with diabetic kidney disease. A uniform balanced cross-over design is used, with the objective to recruit 40 adult participants with diabetic kidney disease, from public and private out-patient settings in French speaking Switzerland. Participants are randomized in equal number into four study arms. Each participant receives usual care alternating with the multidisciplinary self- care management program. Each treatment period lasts three months and is repeated twice at different time intervals over 12 months depending on the cross-over arm. The multidisciplinary self-care management program is led by an advanced practice nurse and adds nursing and dietary consultations and follow-ups, to the habitual management provided by the general practitioner, the nephrologist and the diabetologist. Data is collected every three months for 12 months. Quality of life is measured using the Audit of Diabetes-Dependent Quality of Life scale, patient self-care behavior is assessed using the Revised Summary of Diabetes Self-Care Activities, and adherence to anti-hypertensive therapy is evaluated using the Medication Events Monitoring System. Blood glucose control is measured by the glycated hemoglobin levels and renal function by serum creatinine, estimated glomerular filtration rate and urinary albumin/creatinine ratio. Data will be analyzed using STATA version 14.

**Discussion:**

The cross-over design will elucidate the responses of individual participant to each treatment, and will allow us to better evaluate the use of such a design in clinical settings and behavioral studies. This study also explores the impact of a theory-based nursing practice and its implementation into a multidisciplinary context.

**Trial registration:**

ClinicalTrials.gov identifier: NCT01967901, registered on the 18th of October 2013.

**Electronic supplementary material:**

The online version of this article (doi:10.1186/s12882-016-0279-6) contains supplementary material, which is available to authorized users.

## Background

Diabetic Kidney Disease (DKD) is becoming a global health concern [1] that affects largely the elderly population [2]. Despite advances in pharmacology and management strategies, it remains associated with high morbidity and mortality [3]. Non-adherence to the treatment regimen is thought to be the major cause for the poor control and the complications in these patients [4]. Multidisciplinary management of chronic disease has been shown to improve patient outcomes [5]. Up to our knowledge, few studies addressed specifically the multidisciplinary management of DKD and none evaluated the effect of multidisciplinary self-care management on renal function. A multidisciplinary self-care management program (MSMP) could optimize self-care, and improve the outcomes of patients with DKD.

DKD affects approximately 30 to 40 % of diabetic patients [6, 7]. It is a progressive disease associated with multiple co-morbidities, major complications and increased health costs. Its main complications include renal failure, and cardiovascular complications [8]. The frequent hospitalizations associated with the progression of the disease and dialysis increase considerably the health care costs [9]. Further, decreased kidney function was found to be associated with a highly compromised health related quality of life [10]. The management of DKD aims at improving patient outcomes such as functional status, and quality of life [11], preventing the progression of kidney disease and cardiovascular complications [7]. Patients, with multiple chronic diseases, like DKD, living at home, are expected, on daily basis, to fulfill their own self-care activities [12]. Patients are in general unable to accomplish their self-care effectively without assistance and guidance for acquiring symptom monitoring and interpretation skills, setting priorities and decision making [13]. According to a meta-analysis, chronic disease self-management programs have shown to improve patients’ clinical outcomes, like glycemic control [14]. A recent meta-analysis documented that diabetes mellitus self-management programs may improve the patients quality of life [15]. The use of multidisciplinary management of chronic kidney disease (CKD), have been recommended starting at an early stage of the disease [16]. Several systematic reviews of the literature or meta-analysis confirm the effectiveness of multidisciplinary clinic based nurse-led management programs, multidisciplinary home visit management programs, and multidisciplinary home tele-management programs of various chronic diseases, such as heart failure, diabetes, and kidney disease, in improving one or more patient outcomes such as reducing costs, re-hospitalization rates and mortality, increasing health literacy, and adherence to treatment, ameliorating patients functional status, self- care abilities and quality of life [11, 17–23]. Few of the multidisciplinary chronic disease trials used strategies to improve patient self-care as their primary intervention [24–30]. These studies addressed mainly heart failure patients and did not include home visits in their intervention. One study addressed self-management strategies in the multidisciplinary management of DKD [31]. However, this study did not evaluate the effect of the intervention on kidney function, did not investigate a role for an advanced practice nurse (APN), and did not include home visits. Considering the constant and predicted increase in the aging population, the increasing prevalence of DKD, its clinical significance, and its impact on the daily life of patients and on the health care system, a MSMP is a promising approach for improving patients’ outcomes and stabilizing the kidney function.

## Methods/Design

### Study aim

The present study is designed to evaluate the impact of a MSMP on quality of life, self-care behavior, adherence to anti-hypertensive treatment, glycemic control, and renal function in adults with DKD. The study protocol adheres to the SPIRIT reporting guidelines. A SPIRIT checklist is provided as an additional file (Additional file 1).

The research hypotheses are as follows:

1. The participants with DKD enrolled in the MSMP demonstrate a significant improvement in their quality of life, self-care behavior, adherence to anti-hypertensive therapy, glycemic control, and renal function as compared to the usual standard care.
2. A high self-care behavior is associated with a high quality of life and an improved glycemic control.

### Study design and setting

The study is using a uniform and strongly balanced cross-over design. The cross-over design is recommended for an efficient comparison of treatments when recruiting fewer participants in order to attain the same level of statistical power or precision as a randomized controlled trial. It is for use more importantly in chronic disease where treatment aims at slowing the progression of the disease, improving quality of life and preventing complications. Thus, the patient responses to each treatment are then compared [32], because in cross-over design, each participant will receive the treatment and serve as his own control. Further, a review of randomized control trial (RCT) interventions designed to improve outcomes of patients with multi-morbidity showed that interventions had mixed effects. Patients with chronic diseases enrolled in the RCTs present heterogeneity of co-morbidities, therefore comparisons between two groups of patients seem to be difficult [33]. The strongly balanced and uniform design represents the ideal cross-over design that is able to overcome the statistical bias of the first order carry-over effect and minimize that of the second order [32]. In the present study, we considered the effect of the second order carry-over to be negligible. Each participant is receiving the standard usual care (UC) and the MSMP at different time periods during the course of the trial, as described in Table 1 on “Cross-over plan of participants enrolled in the MSMP study”. This design will allow us to determine the effectiveness of an intensive multidisciplinary follow-up as compared to the standard usual follow-up. The study is being conducted in public and private out-patient settings in the French speaking part of Switzerland. Patients are followed up in the nephrology out-patient service of the public teaching hospital, namely the Centre Hospitalier Universitaire Vaudois (CHUV), in the diabetology out-patient service of a private medical center, namely Clinique La Source, and in private practice.

**Table 1:** Cross-over plan of participants enrolled in the MSMP study

Design	Period 1 0–3 months	Period 2 3–6 months	Period 3 6–9 months	Period 4 9–12 months
Sequence ABBA (*n* = 10)	A = UC	B = MSMP	B = MSMP	A = UC
Sequence BAAB (*n* = 10)	B = MSMP	A = UC	A = UC	B = MSMP
Sequence AABB (*n* = 10)	A = UC	A = UC	B = MSMP	B = MSMP
Sequence BBAA (*n* = 10)	B = MSMP	B = MSMP	A = UC	A = UC

### Study intervention

Patients who have accepted to participate are enrolled in the study for 12 months. Each participant receives UC twice over three months at different time intervals, depending on the cross-over sequence he or she was assigned to. In addition, each participant receives the MSMP twice over three months at different time intervals. Therefore, participants cross-over every three months for a total of three times as described in Table 1 on “Cross-over plan of participants enrolled in MSMP study”.

#### Usual care period-UC

During this period, participants receive the UC management that consists of a follow-up by their general practitioner, nephrologist and/or diabetologist. The participants continue to visit their nephrologist every three months, on average. Other healthcare professionals such as a pharmacist, a physiotherapist, a podiatrist, a psychologist, and a social worker may be consulted as needed. Depending on previous hospitalizations or on earlier referrals made by their general practitioner, or diabetologist, participants might have been exposed, prior to the commencement of the study to diabetes education and, or dietary intervention. However, we assume that they have not been exposed to self-care management programs because the self-care management approach of the current study is a pioneer one in the French speaking part of Switzerland.

#### The multidisciplinary self-care management program period- MSMP

During this period, participants continue to see their general practitioner, usual nephrologist and/or diabetologist. In addition, they receive multidisciplinary care, led and coordinated by an APN. The MSMP care consists of nursing care by a nurse specialized in diabetes care from the out-patient service of a private medical center, namely the Clinique La Source and follow-ups by a private practice dietician. A diabetes specialized nurse is delivering the main nursing intervention because most of the patients’ expected daily self-care activities are related to diabetes management and a nursing knowledge and an expertise in this area is essential for a successful follow-up. Patients who were not followed-up by a diabetologist prior to their enrollment in the study, are systematically referred to a diabetologist resident in the CHUV for one basic consultation. The diabetes specialized nurse and the dietician, adopt as recommended, a self-care management approach that is patient-centered responding to patients’ preferences, needs and values, supporting patients in informed decision-making, and developing problem-solving skills [34]. The program consists of two dietary consultations, three nursing consultations at home and/or at the out-patient clinic, and two nursing telephone follow-ups, under the supervision of the study diabetologist. Participants receive a follow-up every two weeks, by one of the MSMP healthcare member, that is the diabetes specialized nurse, a dietician, a nephrologist or a diabetologist. This frequency was adopted based on the results of a retrospective cohort of patients with diabetes mellitus (*n* = 26,496) that evaluated the effective frequency of contact with a physician that would help achieve the targeted patient clinical outcomes such as glycemic control and blood pressure [35]. The intervention starts with a consultation with the nurse who completes a comprehensive initial assessment and shares it with the multidisciplinary team. An example of the schedule, the description and the evaluation of a MSMP sequence is presented in Table 2 on “The schedule, description and evaluation of the MSMP sequence-BAAB for patients with DKD”. Each nursing consultation takes about one hour, except for the initial consultation that lasts for about one and a half hours. Each nursing telephone follow-up takes about fifteen minutes. At the end of the study each participant would have received at least six nursing face to face follow-ups, four nursing telephone follow-ups, and four face to face dietary follow-ups amounting to a grand total of eleven and a half hours.

**Table 2:** The schedule, description and evaluation of the MSMP sequence-BAAB for patients with DKD

			An multidisciplinary self-management program sequence BAAB								
			Weeks 1 & 41	Weeks 3 & 43	Weeks 5 & 45	Weeks 7 & 47	Weeks 9 & 49	Weeks 11 & 51	Weeks 12 & 52	Weeks 13, 27–28, 39–40, 52–54	Week 52–54
	Assessment and/or Intervention	Medical visit & Screening	Nurse Home Visit	Nurse Teleph. follow-up	Dietician Clinic Visit	Nurse Home Visit	Dietician Clinic Visit	Nurse Telep. Follow-up	Nurse Clinic Visit	Medical Visit nephrologist or diabetologist	End of the study
Enrollment	Inclusion/Exclusion criteria	X									
	Information form	X									
	Randomization	X									
	Informed consent form	X									
Treatment	1. Comprehensive initial assessment & evaluation of patients self-care deficits		X							Medical follow-up	
	Current Medications		X								
	Priority setting-one goal & contract signing		X								
	2. Teaching & Training on self-care		X			X					
	Education on DKD					X					
	Education on the risk of hypoglycemia						X				
	3. Counseling on self-care development					X	X				
	4. Guiding & support			X		X	X				
	5. Coordination of Care		X	X	X	X	X	X	X		
	6. Follow-up & proactive monitoring			X		X		X			
	Dietary plan and counseling				X				X		
Outcomes’ measurements	Demographics		X								
	Self-care behavior		X						X		X
	Medication adherence		X						X		X
	Quality of life		X						X		X
	Serum cr, eGFR, urinary albumin/cr ratio		X						X		X
	HbA1c		X						X		X
	Resource utilization		X						X		X

#### The role of the advanced practice nurse- APN

In this MSMP, an APN manages the program and ensures the coordination of care within the program and among the public and the private sector. This person is a nurse and a PhD nursing candidate. The APN role has been fashioned based on Hamric et al. [36]. The APN is based in the CHUV nephrology service. She coordinates the consultations of the dietician and the diabetes specialized nurse. She provides guidance to the diabetes specialized nurse and the dietician, assisting them in tailoring care based on evidenced-based intervention and safeguarding a self-care management approach. She discusses each participants care plan and goals with the diabetes specialized nurse before and after each consultation. In addition, the APN discusses each care plan and patient progress with the study diabetologist and the participant’s nephrologists. Both of these specialty physicians may discuss patient management with the general practitioner and recommend changes in the medication regimen. The APN also coordinates the participant care with other healthcare professionals such as pharmacists, physiotherapists, and social workers, as needed. Due to the dispersion of the multidisciplinary team between public and private sectors, and in order to facilitate the communication flow and ensure timely sharing of participants care information, needs and progress, an electronic application was developed. This application aimed at sending, directly, the specific assessment and follow-up forms filled by the diabetes specialized nurse to the APN and/or the dietician. Due to this immediate sharing of information, the APN is constantly informed and updated on patient care and the diabetes specialized nurse’s comprehensive assessment is available to the dieticians before her first encounter with the participant. In addition, the APN is responsible for the efficacy of the study, its data collection, and management.

#### The role of the study diabetologist

The multidisciplinary team benefits of a diabetologist who acts as a consultant for the study. This person is a DKD specialist and holds a joint position both in the Service of Diabetes and Endocrinology, and the Service of Nephrology of the CHUV. During the study, she constantly discusses the participants care plan with the APN, as well as with the diabetes specialized nurse and the dietician when necessary. She also discusses participants’ care plan with the respective nephrologist and may recommend a change in the patient’s diabetic medications or antihypertensive regimen.

#### The role of the diabetes specialized nurse

Orem’s Self-Care Deficit Theory as described in the section entitled “the science of the development and exercise of self-care agency” [37], was used to inform the diabetes specialized nurse’s role. Specific evidence-based practice nursing assessment and follow-up forms and educational materials were identified and adapted to the purpose of the study and its specific patient population. All of these forms and materials were translated into French, and approved by the study team. In the MSMP, the diabetes specialized nurse institutes a self-care program. Her role consists of providing a comprehensive initial clinical and psychosocial assessment of the participant, an evaluation of the participant medication safety use, and the development of a care plan collaboratively with the participant who will identify a priority treatment goal. The participant rates his or her confidence of potentially attaining the goal and sign a contract with the diabetes specialized nurse. The diabetes specialized nurse then develops nursing interventions that will help the participant meet the goal. Nursing interventions include tailored teaching, counseling and support. All participants receive evidenced based educational material on how to best protect their kidneys, that was conceived for the purpose of the study. Participants are asked to contact the diabetes specialized nurse during working hours for any question related to the MSMP and the emergency department of the CHUV outside working hours.

#### The role of the dietician

All participants are screened and evaluated by one of the two dieticians of the study. Each receives an individualized dietary care plan. Subsequently, the dietician may recommend to the diabetes specialized nurse to reinforce dietary teaching during nursing follow-ups. The dietician documents the participant’s dietary goal, summarizes the follow-up and communicates it to the diabetes specialized nurse and the APN. The dietician provides the patients with teaching and evidenced based dietary pamphlets developed by the local dietetic association on hyper- and hypoglycemia management, moderate salt diet, potassium content in foods and healthy protein intake.

#### Identification of eligible patients

Patients are recruited with the help of the nephrologists of the CHUV out-patients Nephrology Service and diabetologists in one mid-size town of the French speaking part of Switzerland, according to the inclusion and exclusion criteria described in Table 3 on “Inclusion and exclusion criteria”. The physicians give patients detailed information on the study and are available along with the APN to answer patients’ questions.

**Table 3:** Inclusion and exclusion criteria

Inclusion criteria
1. Age 18 and over.
2. Clinical diagnosis of diabetes.
3. Clinical diagnosis of renal disease and an estimated glomerular filtration rate (eGFR) of less than 60 ml/min calculated based on the CKD Epidemiology Collaboration (CKD_EPI) formula and/or an Albumin/Creatinine ratio of 30 mg/mmol or more.
4. Free of cognitive deficit as assessed by the recruiting physician, based on a normal score on the French version of the Short Portable Mental Status Questionnaire [45]. In the case of a patient being diagnosed with a cognitive deficit, the physician ensures patient’ referral or follow-up.
5. Free of psychomotor skills limitations as determined by the recruiting physician based on the physical examination of the patient.
6. Able to read, write and speak in French.
7. Signed informed consent.
Exclusion criteria
1. Terminal illness other than CKD such as cancer or severe heart failure.
2. Planned major surgical procedures.
3. Patient on dialysis.
4. Patient receiving nursing home care visits for the management of diabetes.

### Randomization

The recruiting diabetologists and nephrologists are blinded to the allocation sequence. The allocation sequence was generated by a computer random number generator, allowing equal numbers of patients in the, study four arms, referred to as sequences of follow-up, as shown in Table 1 on “Cross-over plan of participants enrolled in the MSMP study”. Patients who have accepted to participate and signed the written consent, are referred by the recruiting physician to the APN who assigns them chronologically according to the allocation sequence. After patients are assigned to a sequence, the patients and healthcare providers are no more blinded to the intervention sequence.

### Primary outcome

The primary outcome of the study is quality of life. It is measured using the French version of the Audit of Diabetes-Dependent Quality of life (ADDQoL) measure. The ADDQoL is a self-administered questionnaire constituted of 19 items covering the present quality of life, the impact of diabetes on quality of life, the impact of diabetes on life domains mainly the social life, the physical health, the self-confidence, the motivation feelings about the future, the dependency on others, the living conditions and others (Cronbach α coefficient = 0.947) [38].

### Secondary outcomes

The secondary outcomes, described in Table 4 on the “Overview of the MSMP study outcome variables”, are self-care behavior including adherence to anti-hypertensive treatment, and clinical outcomes, including blood glucose control and renal function. Demographic and clinical data are also collected from participants’ records and interviews using a demographic data questionnaire. The MSMP use outside the defined frequency of the intervention is also assessed. Secondary outcomes are measured as follows:

**Table 4:** Overview of the MSMP study outcome variables

Dependent variables	Dimension (s) measured	Measure used
Patient Variables		
Quality of Life	Present Quality of Life	ADDQoL
	Impact of Diabetes on Quality of Life	ADDQoL
	Impact of Diabetes on Life Domains	ADDQoL
Self-Care Behavior	Diet Habits	R-SDSCA
	Exercise Habits	R-SDSCA
	Blood Sugar Testing	R-SDSCA
	Foot Care	R-SDSCA
	Smoking	R-SDSCA
	Adherence to Antihypertensive Treatment	MEMS
Patient Clinical Variables		
Blood Glucose control	HbA1c	%
Kidney Function	Serum Creatinine	μmol/L
	eGFR	ml/min using CKD_EPI
	Urinary Albumin/Creatinine Ratio	mg/mmol
Resource Utilization Variable		
The MSMP use outside the defined frequency of the intervention	The number of times the participant seek help from the MSMP outside the defined frequency of the intervention	APN Records Nursing Records Dietary Records

1. Patient self-care behavior using the French version of the Revised Summary of Diabetes Self-Care Activities (R-SDSCA) which is a self-administered questionnaire, constituted of 10 items covering dietary habits, physical activity, blood glucose monitoring, foot care, and smoking (mean inter-item = 0.47; except for diet mean inter-item = 0.40) [39].
2. Adherence to anti-hypertensive therapy using the Medication Events Monitoring System (MEMS) [40], throughout the study.
3. Blood glucose control through the measurement of glycated hemoglobin (HbA1c).
4. Kidney function through the measurement of serum creatinine, eGFR using CKD_EPI formula and urinary albumin/creatinine ratio.
5. The MSMP use outside the defined frequency through the calculation of the number of times participants seek help from the MSMP team outside the defined frequency.

### Data collection and management

Participants are enrolled in the study for 12 months. Measurements are carried out after each follow-up period. A total of four measurement times are required; at baseline (T0), at the end of three months of follow-up (T1), at the end of six months of follow-up (T2), at the end of nine months of follow-up (T3) and at the end of 12 months of follow-up (T4).

The laboratory carrying out the analyses of the blood and urine sample is blinded to the study protocol and patient allocation sequence. The self-administered questionnaires are posted to the patient with return envelops or given to the patient to answer while waiting for his appointment with the physician. Reminder letters are sent to patients who fail to respond to the self-administered questionnaires within a week. Data collection is organized and managed by the APN. Laboratory results are entered by the blinded laboratory personnel and double checked by the APN. Results of the self-administered questionnaire are entered by the APN and overseen by a computer assistant, who is blinded to the patient sequence allocation. The statistician carrying out the data analysis is independent from the study and blinded to the patient allocation sequence.

In order to promote participant retention, the recruiting physician, discusses the study with the patient every three months during the regular follow-up. Also, the study is designed with home visits that would facilitate participation of the patients and thus contributing to study retention.

### Statistical analysis

The data will be analyzed, with intent to treat concept, using SPSS for Windows version 22. Data will be expressed as means ± Standard deviation of the mean, and a p-value of less than 0.05 will be considered statistically significant. Statistics that account for the fact that participants serve as their own control will be performed to detect the changes in variables at each follow-up period. Pearson’s product correlation test will be used to detect possible correlations among variables.

### Data monitoring

Data is monitored by the ethics committee who requires adverse events reporting, in addition to a yearly interim reporting on the progress of the study, the processes, and the number of drop-outs.

### Safety assessments

The investigators assume that there will be no risk associated with the study and participants’ psychological and biological integrity will be preserved. Nonetheless, an institutional insurance is obtained from the CHUV. As soon as participants are enrolled in the study, the general practitioner is informed. The study adds an intensive follow-up by a multidisciplinary team and does not alter or interfere with follow-up visits of the general practitioner who continue the participant’s care as planned. At the end of the study, the multidisciplinary team will remain available for follow-up if desired by the general practitioner. The study diabetologist oversees the care plans, ensures participants’ surveillance and will terminate participation if changes in clinical conditions emerge and the participant falls under one or more of the study exclusion criteria.

### Efficacy assessments

In order for the nurse’s role to be possible, clinical reasoning skills need to be present, hence, a nurse with 20 years of experience in the field of diabetes care was recruited to the study. Also, the recruited diabetes specialized nurse believes in the intervention, the importance of fostering self-care, and is confident in being able to execute the nursing role as required by the protocol. The diabetes specialized nurse follows up all patients included in the study and throughout the whole study period. To ensure efficacy and internal consistency, care was structured by using electronic forms for data entry on patient assessment and follow-up. All the specific assessment forms and follow-up forms were transformed into electronic questionnaires. Hence, few questions, relevant to the patient assessment, are mandatory in order to proceed to the following questions. Rendering questions mandatory ensures completeness of the comprehensive patient assessment, without missing information. All the verbal communications within the multidisciplinary team is also documented electronically. Each patient signs with the diabetes specialized nurse, a contract describing his or her priority goal. The contract includes a description of the barriers and the plan for attaining the goal.

### Power calculation

Patients’ quality of life is our main outcome. Multiple chronic diseases are associated with compromised quality of life [41]. Our patient population suffers from two chronic conditions, i.e., diabetes and kidney disease. Quality of life degrades with the continuous decline of the kidney function [42]. However, improvement of patients’ quality of life is one of the main goals of the management of DKD [11]. A recent meta-analysis documented that diabetes self-management programs may improve patients’ quality of life [15] and the American Diabetes Association [34] recommends the measurements of quality of life as an endpoint in diabetes self-care management programs. Considering quality of life as measured by the ADDQoL in patients with complications [43] as the primary outcome for the estimation of a statistical power and a 20 % improvement on the ADDQoL as clinically significant, at an α of 0.05 & a power of 0.08 for powered statistical tests of significance and the variance for all variables, 32 patients are required to detect a 20 % absolute difference with three months intervals. To compensate for a 20 % expected drop-out rate, a total number of 40 participants is required.

### Estimated timeline

The expected study duration is 36 months.

### Confidentiality and data security

Participants’ privacy is respected. Results and personal information are kept confidential. Only the investigators have access to the participants’ data which is coded to ensure confidentiality.

### Retribution

No compensation or retribution for participation in the study is offered. However, the cost of the transportation to the diabetes specialized nurse and the dietician private practice are reimbursed. Blood tests including serum creatinine, eGFR, urinary albumin/creatinine ratio and HbA1c are charged on the study expenses.

## Discussion

Results of the study will show whether a MSMP for patients with DKD improves quality of life and delay the progression of the disease by preserving the kidney function, through optimization of the patient adherence to anti-hypertensive treatment, development of self-care abilities and achievement of an optimal glycemic control. Few studies have employed an experimental prospective design to evaluate multidisciplinary management in DKD to date. Therefore, to elucidate the effect of a multidisciplinary management of DKD, randomized trials are needed. The present study offers to patients with DKD at an early stage of the disease, a six months individualized patient care as part of a multidisciplinary self-care management including clinic and home nursing visits. A balanced cross-over design is used in this study. This design has rarely been used in behavioral and nursing studies. However, cross-over designs are recommended for understanding each patient’s response to different treatments. Thus, it will be possible to better evaluate the efficacy of such designs in clinical settings and behavioral studies. Recruitment from the DKD population is challenging as this population presents multi-morbidities. Adding nursing visits, home visits, dietary visits and telephone follow-ups at a frequency of one every two weeks, is constraining to some patients.

One of the study limitations can be attributed to the role of APN. She delivers the coordination of care as part of the study MSMP intervention and is directly involved in outcome data organization. She is overseeing data collection when not blinded to treatment allocation. However, outcomes measurements were carried out by the participant himself for the self-reported questionnaire and by an independent laboratory for the clinical outcomes. These measures prohibited the APN from influencing data collection directly.

This study will shed the light on an advanced role for nurses in Switzerland. It is also evaluating the impact of integrating a theory based nursing role into practice. The roles of the APN, the diabetes specialized nurse, and the flow of the coordination among all team-members is designed in a way that meets the dynamics of the Swiss healthcare system, while at the same time introducing a new role for nurses. In addition to the usual medically delegated tasks, the nurse has the opportunity to exercise her critical thinking and demonstrate her role in the multidisciplinary team through her assessment, competencies in patient follow-up and coordination of care. One strength of this study is the fostering of collaboration between the public and the private sector in Switzerland. However, this collaboration adds a major challenge to patient care coordination. This is due to the fact that the multidisciplinary team in this study is not based at the same place. Some team members are working in the public sector, some others in the private sector and at differing locations. Therefore, the multidisciplinary team collaboration is “virtual” and proper communication is essential to make up for the lack of a physical setting for encounters and constant exchange. To improve coordination of participants’ visits to different healthcare professionals, the appointments with the dieticians and the diabetes specialized nurse were centralized with the APN. As each of these providers had other patients outside the study to look after, coordination of appointment may present as a limitation. When participants miss an appointment, rescheduling becomes constraining and much exchanging is necessary in order to achieve a successful rescheduling. In an exploratory study, using focus groups with patients with type 2 diabetes mellitus, the lack of individualized and coordinated care were the most often reported perceived barriers to effective implementation of self-care plans [44]. Beyond these difficulties, results of this study will highlight the importance of a multidisciplinary care with self-care management as a primary goal, for ensuring an individualized collaborative approach, in which the patient plays a major role.

## Abbreviations

ADDQoL, audit of diabetes-dependent quality of life; APN, advanced practice nurse; CHUV, Centre Hospitalier Universitaire Vaudois/University Medical Center Vaud; CKD, chronic kidney disease; CKD_EPI, Chronic Kidney Disease Epidemiology Collaboration; DKD, diabetic kidney disease; eGFR, estimated glomerular filtration rate; HbA1c, glycated hemoglobin; MEMS, Medication Events Monitoring System; MSMP, Multidisciplinary self- care management program; RCT, Randomized controlled trails; R-SDSCA, revised summary of diabetes self-care activities questionnaire; UC, usual care

## Additional file

Additional file 1: SPIRIT Checklist. (DOC 123 kb)
